# Breastfeeding in women with juvenile idiopathic arthritis: results from a Norwegian quality register

**DOI:** 10.1186/s12884-025-07570-8

**Published:** 2025-04-17

**Authors:** Tuva Birkeland, Åse Størdal, Carina Gøtestam Skorpen, Hege Svean Koksvik, Marianne Wallenius

**Affiliations:** 1 Faculty of Medicine and Health Sciences, NTNU, Norwegian University of Science and Technology, Trondheim, Norway; 2 Department of Health Sciences, Faculty of Medicine and Health Sciences, NTNU, Norwegian University of Science and Technology, Ålesund, Norway; 3 Department of Rheumatology, Ålesund Hospital, Ålesund, Norway; 4 Norwegian National Network for Pregnancy and Rheumatic Diseases (NKSR), Trondheim University Hospital, Trondheim, Norway; 5 Department of Neuromedicine and Movement Science, NTNU, Norwegian University of Science and Technology, Trondheim, Norway

**Keywords:** Juvenile idiopathic arthritis (JIA), Arthritis, Childhood arthritis, Breastfeeding, Self-rated health, Disease-Modifying Antirheumatic Drugs (DMARDs), Corticosteroids, Register study, Women’s health

## Abstract

**Background:**

Limited research exists on the challenges women diagnosed with juvenile idiopathic arthritis (JIA) can face during pregnancy and breastfeeding, and if breastfeeding affects disease related factors.

**Aims and objectives:**

This study aimed to explore the proportion of women with JIA breastfeeding at six weeks, six months, and 12 months postpartum, as well as examining demographic and disease related factors and the use of medications, comparing the breastfeeding and the non-breastfeeding groups.

**Methods:**

Data on women with JIA regarding pregnancy and breastfeeding were collected prospectively from the Norwegian nationwide quality register RevNatus in this observational study. The data included demographics, disease activity, self-reported health status, medication, obstetric and neonatal outcome, and were collected from clinical documentation and self-reported material during visits at the outpatient clinic between January 2016 and July 2023. In this study, we used data from the inclusion visit and from the follow-up at six weeks, six months and 12 months postpartum.

**Results:**

Amongst 304 births in 227 women, 86% of the patients were breastfeeding at six weeks, 70% at six months and 39% at 12 months postpartum. Breastfeeding women differed from non-breastfeeding women in several aspects. At six weeks postpartum, 79% of breastfeeding women had a higher education level compared to 49% of non-breastfeeding women (*p*-value < 0.001). Additionally, breastfeeding women experienced longer pregnancy duration (40 weeks versus 38 weeks, *p*-value 0.004), had a lower prevalence of cesarean section (CS) (21% versus 45%, *p*-value 0.007) and premature birth (5% versus 22%, *p*-value < 0.001), and gave birth to newborns with a mean higher birth weight (3512 g versus 3175 g, *p*-value 0.011). In terms of health status, the breastfeeding women had lower Visual Analog Scale (VAS) scores for pain (24 compared to 38 mm, *p*-value 0.002), fatigue (25 compared to 40 mm, *p*-value 0.030) and total (29 compared to 38 mm, *p*-value 0.023) six weeks postpartum. At all registrations, a larger proportion of non-breastfeeding women used conventional synthetic disease-modifying antirheumatic drugs (csDMARDs) (at six weeks: 29% compared to 21%, *p*-value 0.021; at six months: 30% compared to 27%, *p*-value 0.002; at twelve months: 38% compared to 30%, *p*-value < 0.001).

**Conclusion:**

In the present study, we observed a high proportion of women with JIA breastfeeding at six weeks and six months postpartum. Based on our findings, health professionals should encourage women with JIA to breastfeed when taking compatible medications.

## Background

Juvenile idiopathic arthritis (JIA) is an umbrella term covering a heterogenous group of arthritis that persists for at least six weeks before the age of 16. Approximately half of patients diagnosed with JIA have active disease in adulthood [1, 2] and over 40% of women with JIA in childbearing age either have active disease or are on medications [3]. 

New treatment options over the past 20 years have significantly improved the management of JIA, enabling more women with JIA to successfully have children [4]. Greater understanding of immune-modulating treatments during pregnancy and breastfeeding contributed to this progress [4].

Several disease-modifying antirheumatic drugs are considered to be safe to use when breastfeeding, and mothers with inflammatory rheumatic diseases are advised to breastfeed according to updated international guidelines [5–7]. Nevertheless, we know little about whether these women adhere to the recommendations. The Norwegian Mother, Father, and Child Cohort (MoBa) study from 2010 showed that, in the general population, 80% of six months old infants in Norway were breastfed [8]. To our knowledge, no studies have yet explored the breastfeeding rates among mothers with JIA, and research on the impact of pregnancy on JIA disease activity remains limited. One Norwegian study collected data from 2006 to 2015, including 135 pregnancies in 114 women with JIA, and found that almost 80% of the women were in remission or had low disease activity during pregnancy and postpartum [3]. A Swedish study from 2017 found increased risks of obstetric complications among women with JIA [9], while two Asian studies have concluded with limited adverse obstetrical and neonatal outcomes in JIA pregnancies [10, 11]. A study conducted in Norway in 2023 concluded that the rate of caesarean sections was higher among women with JIA compared to the general population [12].

## Aims and objectives

This study aimed to evaluate the proportion of women with JIA who breastfed at six weeks, six months and 12 months postpartum. It also compared the demographic characteristics, lifestyle factors, disease activity, pregnancy outcomes, and the use of immune-modulating medications among breastfeeding and non-breastfeeding women.

## Methods

We used data from the nationwide quality register RevNatus, which is a consent-based register collecting data about women with inflammatory rheumatic diseases throughout pregnancy and up to 12 months postpartum [13]. All patients aged 16 and older and planning pregnancy, are invited to enroll in the registry. The data are collected from clinical documentation and self-reported material from ordinary visits at the out-patients clinic. A total of seven visits and registrations are conducted during the observation period: one before pregnancy, one in each trimester, and follow-ups at six weeks, six and 12 months postpartum. In this study, we used data from the inclusion visit as well as from the follow-up visits at six weeks, six months and 12 months postpartum. We included all pregnancies in women diagnosed with JIA that led to a live birth and who additionally attended at least the postpartum control assessment at six weeks.

The main categories of variables collected in this study included demographic data, use of stimulants (tobacco and snuff), body mass index (BMI), physical activity (aerobic exercise for at least 30 min once a month), disease-related factors, medications, selected information about the pregnancy and newborn, self-reported data (VAS), breastfeeding at six weeks, six months and 12 months postpartum and postpartum contraception use. Medications were grouped as conventional synthetic DMARDs (csDMARDs), biological DMARDs (bDMARDs) and corticosteroids. Breastfeeding was registered comprehensively without differentiation between exclusive or partial breastfeeding. Demographic data included age, educational level and work status.

Disease-related factors included the duration of the disease, the presence or absence of erosions, and serological results. The serological tests included anti-cyclic citrullinated peptide (anti-CCP) and rheumatoid factor (RF). A disease activity score was calculated using the Disease Activity Score-28 with C-reactive protein (DAS28-CRP-3). To calculate this score, the number of swollen and tender joints among the 28 evaluated joints was recorded. Additionally, the C-reactive protein (CRP) was measured, and the score was calculated using a specific formula [14]. The DAS28-CRP-3 score ranges from 1 to 10. The European Alliance of Associations for Rheumatology (EULAR) has classified disease activity into four categories based on specific cut-offs within the DAS-28-CRP-3 score [14]. These categories are remission (DAS-28-CRP-3 < 2.6), low disease activity (2.6 ≤ DAS-28-CRP-3 ≤ 3.2), moderate disease activity (3.2 < DAS-28-CRP-3 ≤ 5.1) and high disease activity (DAS-28-CRP-3 > 5.1). An improvement in the DAS-28-CRP-3-score of ≥ 1.2 is considered clinically relevant [14].

In the self-reported data, we used visual analogue scales (VAS), a tool to assess the patient’s subjective experience. The patient is presented a 100 mm line, labelled with descriptors such as “no pain/fatigue” at the left end and “worst imaginable pain/fatigue” or an equivalent at the right end, and is asked to mark the level of pain or fatigue, which were scored separately. The VAS total represents the patient’s overall subjective evaluation of the burden of the disease.

Information about pregnancy included maternal age at birth, parity, and selected pregnancy outcomes. Data pertaining to pregnancy outcomes were collected during the visit six-week postpartum visit. These included the date of birth, term date, gestational age, birth weight and the method of delivery (vaginal, vaginal instrumental or caesarian section, whether acute or elective). Additionally, data on birth complications was collected, which included pre-eclampsia, eclampsia, and Hemolysis, Elevated Liver enzymes and Low Platelet syndrome (HELLP) syndrome. Preterm birth was defined as birth before 37 completed weeks of gestation.

### Statistical analysis

Statistical analyses were performed using IBM SPSS Statistics version 29, a software package used for management and statistical analysis of data. The statistical significance level was defined as two-sided *p*-value ≤ 0.05 with no adjustment made for multiple comparisons. Values were expressed as mean ± standard deviation (SD). For group differences between breastfeeding and non-breastfeeding groups for normally distributed and continuous data we used the Independent Samples t-test. For continuous variables not following a normal distribution we used the Mann-Whitney U test to estimate the *p*-value. For categorical variables we used the Pearson chi squared test, or the Fisher´s exact test for small-sample sizes [15].

## Results

**Fig. 1:**
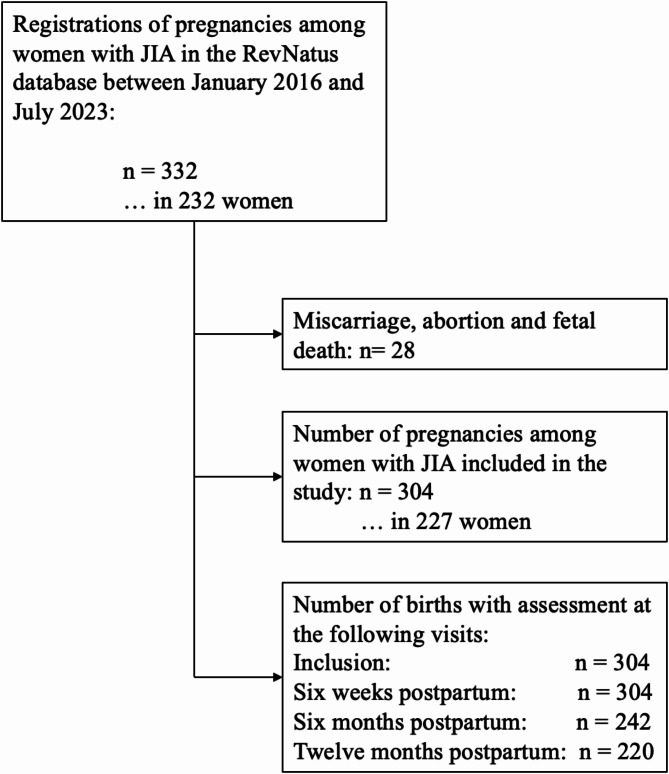
Flow chart depicting the inclusion and exclusion criteria for women in the study

Figure 1 illustrates the flowchart depicting the inclusion and exclusion criteria for women in the study. Between January 2016 and July 2023, 332 pregnancies in 232 women with JIA were registered in RevNatus. The pregnancies leading to miscarriage, abortion, fetal death, stillbirth, and no live birth were excluded. In total, 227 women were included in the study, and 70 of them were registered with more than one pregnancy. This left us with 304 births in 227 women. Not every woman attended every postpartum registration, and not all information was consistently recorded during each registration, resulting in missing data. Out of 304 births, 242 women attended the six-month postpartum follow-up and 220 attended the 12-month postpartum follow-up. Some women in the study are counted multiple times, as they have given birth to more than one child, with each pregnancy being registered separately.

Table 1 shows the background and relevant disease characteristics among women with JIA at inclusion. The mean CRP value was 6 mg/L, and 110 women (39%) had a CRP value above 5 mg/L. The majority of women (68%) were categorized to be in remission according to the DAS-28-CRP-3 score. The mean VAS pain score was 25.3 mm, the mean VAS fatigue score was 41.9 mm, and the mean VAS total score was 29.7 mm. The most frequently prescribed medication were bDMARDs, used by 94 women (31%), with 90 of them recieving a TNF-inhibitor.

**Table 1:** Background and disease characteristics among women with JIA at inclusion

Background characteristics	*N*	
Age (years), mean (SD)	304	28.8 (4.3)
Age > 35 years, *n* (%)		23 (7.6)
Parity, *n* (%)	304	
0		136 (44.7)
1		98 (32.2)
≥2		70 (23.0)
Cigarette/Snuff use *n* (%)	299	30 (10.0)
BMI (kg/m^2^), mean (SD)	293	25.0 (4.4)
Underweight (< 18.5 kg/m^2^), *n* (%)		8 (2.7)
Normal weight (18.5–24.9 kg/m^2^), *n* (%)		164 (56.0)
Overweight ≥ 25 kg/m^2^), *n* (%)		121 (41.3)
Regular physical activity, *n* (%)^a^	201	124 (61.7)
Education level, *n* (%)^b^	298	
Low		10 (3.4)
Intermediate		68 (22.8)
High		220 (73.8)
Working full time or part time, *n* (%)	298	218 (73.2)
**Disease related characteristics**		
Disease duration in years, mean (SD)	269	21.3 (15.3)
Erosive disease, *n* (%)	251	95 (37.8)
Anti-CCP antibodies present, *n* (%)	251	34 (13.5)
RF present, *n* (%)	237	34 (14.3)
CRP (mg/L), mean (SD)	281	6.0 (10.2)
CRP value > 5 mg/L, *n* (%)		110 (39.1)
DAS-28-CRP-3, mean (SD)	253	2.4 (0.8)
Remission (DAS-28CRP-3 < 2.6), *n* (%)		172 (68.0)
Low disease activity (2.6 ≤ DAS-28-CRP-3 ≤ 3.2), *n* (%)		46 (18.2)
Moderate disease activity (3.2 < DAS-28-CRP-3 ≤ 5.1), *n* (%)		34 (13.4)
High disease activity (DAS-28-CRP-3 > 5.1), *n* (%)		1 (0.4)
VAS (mm), mean (SD)		
Pain	228	25.2 (23.8)
Fatigue	217	41.9 (29.5)
Total	224	29.7 (26.2)
**Medications**		
csDMARDs, *n* (%)^c^	302	68 (22.5)^c^
Hydroxychloroquine, *n* (%)		17 (5.6)
Methotrexate, *n* (%)		14 (4.6)
Sulfasalazine, *n* (%)		38 (12.6)
bDMARDs, *n* (%)	303	94 (31.0)
TNF inhibitor, *n* (%)		90 (29.7)
IL-6 inhibitor, *n* (%)		4 (1.3)
Corticosteroids, *n* (%)	303	29 (9.6)

a Aerobic exercise for a minimum of 30 min at least once a month

b Education level: low = elementary school, medium = high school/vocational education, high = college/university

c One woman registered was using both hydroxychloroquine and methotrexate simultaneously

As shown in Table 2, 248 women (85.5%) were breastfeeding at six weeks postpartum, 166 women (70%) at six months, and 80 (39.0%) at 12 months. Table 3 illustrates the proportion of breastfeeding women among primiparous and multiparous women. Multiparous women had the highest breastfeeding rates at both six weeks and six months postpartum. At 12 months postpartum, significantly more primiparous women were breastfeeding compared to multiparous women. Of the primiparous women, 43 (53.8%) were still breastfeeding, while 37 (46.3%) of the multiparous women continued breastfeeding.

**Table 2:** Breastfeeding status at each point of registration

Time of postpartum registration	*N*	Missing	Breastfeeding	Not breastfeeding
Six weeks, *n* (%)	290	14	248 (85.5)	42 (14.5)
Six months, *n* (%)	237	5	166 (70.0)	71 (30.0)
12 months, *n* (%)	205	15	80 (39.0)	125 (61.0)

**Table 3:** Proportion of breastfeeding primiparous and multiparous women at each registration

Time of postpartum registration, *n* (%)	Primiparous	Multiparous	*p*-value^a^
Six weeks, *n* (%)	105 (42.3)	143 (57.7)	0.057
Six months, *n* (%)	70 (42.2)	96 (57.8)	0.638
12 months, *n* (%)	43 (53.8)	37 (46.3)	0.033

a Comparison between groups with Continuity Correction/Chi Square

At six weeks postpartum (Table 4), we observed that women who were breastfeeding had significantly higher levels of education and longer duration of pregnancy. The proportion of premature births and caesarean deliveries was significantly higher among non-breastfeeding women, as was the use of tobacco. Breastfeeding women gave birth to newborns with a higher birth weight and reported significantly less pain, fatigue, and overall disease burden. While breastfeeding women had a significantly lower usage of csDMARDs compared to non-breastfeeding women, there was no significant difference in the use of bDMARDs and corticosteroids.

**Table 4:** Differences in breastfeeding and non-breastfeeding mothers with JIA six weeks postpartum

	*N*	Breastfeeding *N* = 248	*N*	Non-breastfeeding *N* = 42	*p*-value^a^
**Patient background**					
Age (years), mean (SD)	248	29.9 (4.2)	42	28.7 (5.7)	0.119
Age > 35 years, *n* (%)		22 (8.9)		6 (14.3)	0.264
BMI (kg/m^2^), mean (SD)	240	24.9 (4.5)	40	25.2 (3.3)	0.662
Overweight, *n* (%)		104 (43.3)		16 (40.0)	1.000
Working full time or part time, *n* (%)	247	33 (13.4)	39	1 (2.6)	0.061
Regular physical activity, *n* (%)^b^	169	73 (43.2)	28	12 (42.9)	1.000
Cigarette/Snuff use, *n* (%)	241	11 (4.6)	37	5 (13.5)	0.046
Education level, *n* (%)^c^	243		41		< 0.001
Low		5 (2.1)		4 (9.8)	
Intermediate		47 (19.3)		17 (41.5)	
High		191 (78.6)		20 (48.8)	
**Pregnancy and labour**					
Pregnancy duration (weeks), mean (SD)	248	39.5 (1.6)	41	37.9 (3.2)	0.004
Premature birth, *n* (%)^d^		12 (4.8)		9 (22.0)	< 0.001
Birth weight (grams), mean (SD)	247	3511.6 (501.3)	42	3175 (793.3)	0.011
CS, *n* (%)	246	51 (20.7)	42	19 (45.2)	0.007
Emergency CS, *n* (%)		38 (15.4)		13 (31.0)	
Complications, *n* (%)^e^	236	7 (3.0)	41	4 (9.8)	0.063
**Disease related characteristics**					
Disease duration (years), mean (SD)	217	22.1 (16.4)	38	17.7 (9.2)	0.087
CRP(mg/L), mean (SD)	193	6.1 (11.6)	36	4.2 (8.1)	0.067
CRP > 5 mg/L, *n* (%)		60 (31.1)		5 (13.9)	0.057
DAS-28-CRP-3, mean (SD)	171	2.6 (2.3)	32	2.5 (1.1)	0.64
Remission (DAS-28-CRP-3 < 2.6), *n* (%)		110 (64.3)		22 (68.8)	0.78
Active disease (DAS-28-CRP-3 ≥ 2.6), *n* (%)		61 (35.7)		10 (31.3)	
VAS score (mm), mean (SD)					
Pain	190	24.3 (23.8)	34	37.9 (24.9)	0.002
Fatigue		25.4 (24.4)		39.5 (31.9)	0.030
Total	187 191	28.9 (27.3)	33 34	38.4 (26.0)	0.023
**Medications**					
csDMARDs, *n* (%)	247	52 (21.1)	42	12 (28.6)^f^	0.021
Hydroxychloroquine, *n* (%)		14 (5.7)		3 (7.1)	
Methotrexate, *n* (%)		2 (0.81)		4 (9.5)	
Sulfasalazine, *n* (%)		36 (14.6)		6 (14.3)	
bDMARDs, *n* (%)	248		42		0.686
TNF inhibitor, *n* (%)		61 (24.6)		11 (26.2)	
IL-6 inhibitor, *n* (%)		4 (1.6)		1 (2.4)	
Corticosteroids, *n* (%)	247	26 (10.5)	42	4 (9.5)	1.000

a Two-sided *p*-value ≤ 0.05 with no adjustment made for multiple comparisons. Group comparisons were performed using Independent samples t-test for continuous, normally distributed variables. For continuous, ordinal (non-normally distributed) variables, the Mann Whitney U test was performed. Chi Square test was performed for categorical variables, and Fisher´s exact test for small-sample sizes

b Aerobic exercise for a minimum of 30 min at least once a month

c Education level: low = elementary school, medium = high school/vocational education, education, high = college/university

d Birth before gestational week 37

e Preeclampsia, eclampsia, HELLP-syndrome

f One woman registered was using both hydroxychloroquine and methotrexate simultaneously

At the six-month postpartum visit (Table 5), non-breastfeeding women reported higher levels of fatigue compared to breastfeeding women. Additionally, the breastfeeding group had a higher educational level and a lower use of csDMARDs than the non-breastfeeding group (p 0.002). The non-breastfeeding group also had a higher prevalence of tobacco use.

**Table 5:** Differences in breastfeeding and non-breastfeeding mothers with JIA six months postpartum

	*N*	Breastfeeding *N* = 166	*N*	Non-breastfeeding *n* = 71	*p*-value^a^
**Patient background**					
Age (years), mean (SD)	166	30.0 (4.1)	71	28.8 (4.9)	0.077
Age > 35 years, *n* (%)		14 (8.4)		8 (11.3)	0.657
BMI (kg/m^2^), mean (SD)	152	25.9 (15.3)	63	25.6 (4.7)	0.135
Overweight ≥ 25 mg/m^2^), *n* (%)		58 (38.2)		29 (46.0)	0.614
Working full time or part time, *n* (%)	164	14 (8.5)	69	8 (11.6)	0.629
Regular physical activity, *n* (%)^b^	120	72 (60.0)	46	29 (63.0)	0.856
Cigarette/Snuff use, *n* (%)	160	7 (4.4)	66	11 (16.7)	0.005
Education level, *n* (%)^c^	162		70		< 0.001
Low		3 (1.9)		2 (2.9)	
Intermediate		26 (16.0)		29 (41.4)	
High		133 (82.1)		39 (55.7)	
**Disease related characteristics**					
CRP (mg/L), mean (SD)	137	4.5 (7.4)	53	5.0 (5.9)	0.128
CRP value > 5 mg/L, *n* (%)		30 (21.9)		18 (34.0)	0.126
DAS-28-CRP-3, mean (SD)	121	2.7 (2.7)	52	2.5 (0.9)	0.412
Remission (DAS-28-CRP-3 < 2.6), *n* (%)		76 (62.8)		29 (55.8)	0.484
Active disease (DAS-28-CRP-3 ≥ 2.6), *n* (%)		45 (37.2)		23 (44.2)	0.484
VAS-score (mm), mean (SD)					
Pain	130	26.3 (23.4)	53	29.9 (25.1)	0.475
Fatigue	131	30.5 (28.6)		42.8 (31.6)	0.028
Total	128	28.5 (25.4)	48 55	29.6 (25.7)	0.839
**Medications**					
csDMARDs, *n* (%)	162	44 (27.2)	70	21 (29.6)^d^	0.002
Hydroxychloroquine, *n* (%)		9 (5.6)		7 (10.0)	
Methotrexate, *n* (%)		4 (2.5)		10 (14.3)	
Sulfasalazine, *n* (%)		31 (19.1)		5 (7.1)	
bDMARDs, *n* (%)	163		70		0.102
TNF inhibitor, *n* (%)		71 (43.6)		33 (47.1)	
IL-6 inhibitor, *n* (%)		1 (0.61)		3 (4.3)	
Corticosteroids, *n* (%)	164	21 (12.8)	70	10 (14.3)	0.924

a Two-sided *p*-value ≤ 0.05 with no adjustment made for multiple comparisons. Group comparisons were performed using Independent samples t-test for continuous, normally distributed variables. For continuous, ordinal (non-normally distributed) variables, the Mann Whitney U test was performed. Chi Square test was

b performed for categorical variables, and Fisher´s exact test for small-sample sizes

c Aerobic exercise for a minimum of 30 min at least once a month

d Education level: low = elementary school, medium = high school/vocational, education, high = college/university

e One woman was registered using both hydroxychloroquine and methotrexate simultaneously

The only significant finding at the 12-month postpartum visit (Table 6) was related to the use of csDMARDs. 30% of breastfeeding women were using csDMARDs, compared to 38% in the non-breastfeeding group (*p* < 0.001).

**Table 6:** Differences in breastfeeding and non-breastfeeding mothers with JIA 12 months postpartum

	*N*	Breastfeeding *N* = 80	*N*	Non-breastfeeding *N* = 125	*p*-value^a^
**Patient background**					
Age (years), mean (SD)	80	30.2 (4.2)	125	29.5 (4.7)	0.231
Age > 35 years, *n* (%)		9 (11.3)		13 (10.4)	1.000
BMI (kg/m^2^), mean (SD)	76	24.2 (4.5)	110	25.2 (4.8)	0.131
Overweight ≥ 25 kg/m^2^), *n* (%)		29 (38.2)		44 (40.0)	0.460
Working full time or part time, *n* (%)	79	50 (63.3)	123	63 (51.2)	0.123
Regular physical activity, *n* (%)^b^	64	33 (51.6)	84	50 (59.5)	0.424
Cigarette/Snuff use, *n* (%)	78	5 (6.4)	120	18 (15.0)	0.073
Education level, *n* (%)^c^	78		122		0.056
Low		2 (2.6)		5 (4.1)	
Intermediate		11 (14.1)		34 (27.9)	
High		65 (83.3)		83 (68.0)	
**Disease related characteristics**					
CRP (mg/L), mean (SD)	67	4.2 (5.5)	110	4.9 (6.4)	0.226
CRP value > 5 mg/L, *n* (%)		17 (25.4)		32 (29.1)	0.717
DAS-28-CRP-3, mean (SD)	60	2.4 (0.8)	101	2.5 (0.8)	0.224
Remission (DAS-28-CRP-3 < 2.6), *n* (%)		40 (66.7)		58 (57.4)	0.32
Active disease (DAS-28-CRP-3 ≥ 2.6), *n* (%)		20 (33.3)		43 (42.6)	0.32
VAS-score (mm), mean (SD)					
Pain	71	26.7 (24.0)	98	33.8 (33.4)	0.281
Fatigue		33.5 (27.4)	93	38.6 (29.0)	0.324
Total	70 67	31.4 (26.8)	98	34.7 (29.1)	0.689
**Medications**					
csDMARDs, *n* (%)	80	24 (30.0)	123	47 (38.2)^d^	< 0.001
Hydroxychloroquine, *n* (%)		2 (2.5)		10 (8.1)	
Methotrexate, *n* (%)		1 (1.3)		23 (18.7)	
Sulfasalazine, *n* (%)		21 (26.3)		15 (12.2)	
bDMARDs, *n* (%)	80		125		0.396
TNF inhibitor, *n* (%)		35 (43.8)		63 (50.4)	
IL-6 inhibitor, *n* (%)		1 (1.25)		4 (3.2)	
Corticosteroids, *n* (%)	80	16 (20.0)	125	17 (13.6)	0.322

a Two-sided *p*-value ≤ 0.05 with no adjustment made for multiple comparisons. Group comparisons were performed using Independent samples t-test for continuous, normally distributed variables. For continuous, ordinal (non-normally distributed) variables, the Mann Whitney U test was performed. Chi Square test was

b performed for categorical variables, and Fisher´s exact test for small-sample sizes

c Aerobic exercise for a minimum of 30 min at least once a month

d Education level: low = elementary school, medium = high school/vocational, education, high = college/university

e One woman registered was using both hydroxychloroquine and methotrexate simultaneously

## Discussion

At six weeks postpartum, 86% of the women in the current study were breastfeeding. This proportion decreased to 70% at six months and 39% at 12 months postpartum. Primiparous women had the longest duration of breastfeeding. The proportion of breastfeeding women in our study is consistent with the breastfeeding rates reported in the general population in a nationwide Norwegian study from 2013, which reported rates of 91% at six weeks, 71% at six months and 35% at 12 months postpartum [16].

The significantly higher educational level among breastfeeding women may be attributed to the tendency of more educated women to follow official guidelines or their greater awareness of the benefits of breastfeeding.

Our study demonstrated an association between duration of pregnancy and breastfeeding, wherein pregnancy duration was significantly longer among the breastfeeding women. This can be explained by the fact that premature births are often associated with delays in the first breastfeeding session and latching difficulties, making it difficult to initiate breastfeeding [17]. This is supported by the significantly higher rate of premature births and C-sections among the non-breastfeeding women. A 2016 American study in a healthy population found similar results, showing that children who are born late preterm and early term had lower breastfeeding rates compared to those born full-term or post-term [17]. A Swedish study from 2017 identified increased risk of obstetric complications, such as early-onset pre-eclampsia, preterm birth, and infants born small of gestational age (SGA) among women with JIA [9]. However, it is essential to note that the Swedish study collected data from births between 1992 and 2011. A Norwegian study from 2024 found that preterm birth was more frequent in women with active JIA compared to a healthy control group [18]. Over the past 10 years, knowledge about medications compatible with pregnancy has expanded, particularly regarding the use of TNF-inhibitors [6, 19]. This may be reflected in the results from the Swedish study where patients were included before TNF-inhibitors were recommended in pregnancy. Two Asian studies reported limited adverse obstetrical and neonatal outcomes among pregnant women with JIA, but are of uncertain relevance due to the absence of information regarding medication use during pregnancy, and disparities in pregnancy and maternity care compared to Scandinavia [10, 11].

All VAS scores (pain, fatigue and total) were significantly lower among breastfeeding women at six weeks postpartum, and the VAS fatigue score was significantly lower at six months. This finding may indicate a relationship between breastfeeding and improved self-reported health status. Another possible explanation could be that the women experiencing less pain and fatigue levels had more energy available to breastfeed their child. There was no statistically significant difference in inflammatory activity, as measured by CRP and DAS-28-CRP-3, between the groups. However, there was a higher proportion of women with active disease among those who did not breastfeed at six and 12 months postpartum, which could be a contributing factor to discontinue breastfeeding. The non-breastfeeding group had a significantly higher use of csDMARDs, especially methotrexate. This may be due to the contraindication of methotrexate during breastfeeding, and the uncertainty some patients and doctors experience about using other csDMARDs while breastfeeding– resulting in discontinuation of breastfeeding– or the reinstatement of methotrexate because the women had already stopped breastfeeding for other reasons.

Twelve months postpartum, most women were not breastfeeding, and we also observed a significantly higher use of csDMARDs among the non-breastfeeding women. This could also be due to the uncertainties around methotrexate and other csDMARDs with breastfeeding. Another possible explanation is that, by 12 months post-partum, it is normal to discontinue breastfeeding, as indicated by the results from the nationwide Norwegian study in 2013 [16]. Therefore, women may have started using csDMARDs because they have stopped breastfeeding.

The strength of this study is its prospective design, which tracks a wide range of variables over time. This design reduces the risk of bias, as the outcomes are measured after the exposure, allowing for a better understanding of cause and effect. Another strength of this study is the large population size, with 332 registered pregnancies. The population is homogenous, as all women were diagnosed and followed by a rheumatologist within the same healthcare system, and share a common cultural background where breastfeeding is actively encouraged for its recognized health benefits.

A limitation of this study is the lack of complete data, as not all women attended every follow-up visit, and some did not complete the digital self-reporting at each post-partum assessment. Missing data increases the risk for type II errors, potentially leading to results with low statistical significance, which should be interpreted with caution. The absence of a control group for comparison is another limitation of the study, making it difficult to consider confounding variables. The comparison with results from a healthy control group could only be made indirectly, and the data from the general population dates back to 2013 [16]. Furthermore, there were no other studies performed which specifically focused on breastfeeding women with JIA to provide a direct comparison, making it challenging to determine whether some of the results are random or reproducible. The relative proportion of formula use was not considered in this study. The Norwegian guidelines recommend exclusive breastfeeding for the first six months of life, followed by a combination of breastfeeding and the introduction of solid foods. Norway has an infant healthcare program that provides free health check-ups from children aged 0–5 years [20]. During these consultations, women are offered breastfeeding support (if needed), and a high proportion of mothers exclusively breastfeed their children during the initial six months.

## Conclusion

In the present study, we evaluated breastfeeding rates among women with JIA at six weeks, six months, and 12 months postpartum. At six weeks postpartum, 86% of the women with JIA were breastfeeding. Breastfeeding women had significantly higher educational levels, longer pregnancy durations, a lower prevalence of caesarean sections, as well as lower scores for pain, fatigue and total VAS. Additionally, a lower proportion of breastfeeding women used csDMARDs compared to non-breastfeeding women. At six months postpartum, 70% of women were breastfeeding, and after 12 months, 39% of the women continue to breastfeed. Our results are consistent with breastfeeding rates in the general population. When taking compatible medications, women with JIA should be actively encouraged by healthcare professionals to breastfeed.

## Data Availability

The data is collected from RevNatus, a Norwegian nationwide consent-based quality register. The data cannot be shared publicly due to the requirements of the involved register holders and the general data protection regulation, to protect the privacy of individuals.

## Abbreviations

Anti-CCP: Anti-cyclic citrullinated peptide
bDMARDs: biological Disease Modifying Antirheumatic Drugs
BMI: Body mass index
CS: Cesarean section
CRP: C-reactive protein
csDMARDs: Conventional synthetic Disease Modifying Antirheumatic Drugs
DAS-28-CRP-3: Disease Activity Score-28-CRP
DMARDs: Disease-modifying Antirheumatic Drugs
EULAR: European Alliance of Associations for Rheumatology
HELLP: Hemolysis, Elevated Liver enzymes and Low Platelet syndrome
IL: Interleukin
JIA: Juvenile idiopathic arthritis
MoBA: Norwegian Mother, Father and Child Cohort Study
RF: Rheumatoid factor
SD: Standard Deviation
SGA: Small for Gestational Age
TNFi: Tumor necrosis factor inhibitors
VAS: Visual analogue scale
